# Illness recognition, decision-making, and care-seeking for maternal and newborn complications: a qualitative study in Sarlahi District, Nepal

**DOI:** 10.1186/s41043-017-0123-z

**Published:** 2017-12-21

**Authors:** Tsering P. Lama, Subarna K. Khatry, Joanne Katz, Steven C. LeClerq, Luke C. Mullany

**Affiliations:** 1 Department of International Health, Johns Hopkins Bloomberg School of Public Health, 615 N. Wolfe Street, W5009, Baltimore, MD 21205 USA; 2 Nepal Nutrition Intervention Project—Sarlahi (NNIPS), Kathmandu, Nepal

**Keywords:** Maternal mortality, Neonatal mortality, Maternal complications, Newborn complications, Illness recognition, Care-seeking, Nepal

## Abstract

**Background:**

Identification of maternal and newborn illness and the decision-making and subsequent care-seeking patterns are poorly understood in Nepal. We aimed to characterize the process and factors influencing recognition of complications, the decision-making process, and care-seeking behavior among families and communities who experienced a maternal complication, death, neonatal illness, or death in a rural setting of Nepal.

**Methods:**

Thirty-two event narratives (six maternal/newborn deaths each and 10 maternal/newborn illnesses each) were collected using in-depth interviews and small group interviews. We purposively sampled across specific illness and complication definitions, using data collected prospectively from a cohort of women and newborns followed from pregnancy through the first 28 days postpartum. The event narratives were coded and analyzed for common themes corresponding to three main domains of illness recognition, decision-making, and care-seeking; detailed event timelines were created for each.

**Results:**

While signs were typically recognized early, delays in perceiving the severity of illness compromised prompt care-seeking in both maternal and newborn cases. Further, care was often sought initially from informal health providers such as traditional birth attendants, traditional healers, and village doctors. Key decision-makers were usually female family members; husbands played limited roles in decisions related to care-seeking, with broader family involvement in decision-making for newborns. Barriers to seeking care at any type of health facility included transport problems, lack of money, night-time illness events, low perceived severity, and distance to facility. Facility care was often sought only after referral or following treatment failure from an informal provider and private facilities were sought for newborn care. Respondents characterized government facility-based care as low quality and reported staff rudeness and drug type and/or supply stock shortages.

**Conclusion:**

Delaying the decision to seek skilled care was common in both newborn and maternal cases. Among maternal cases, delays in receiving appropriate care when at a facility were also seen. Improved recognition of danger signs and increased demand for skilled care, motivated through community level interventions and health worker mobilization, needs to be encouraged. Engaging informal providers through training in improved danger sign identification and prompt referral, especially for newborn illnesses, is recommended.

**Electronic supplementary material:**

The online version of this article (10.1186/s41043-017-0123-z) contains supplementary material, which is available to authorized users.

## Background

Maternal mortality ratio in Nepal has decreased from an estimated 901 to 258 maternal deaths per 100,000 livebirths from 1990 to 2015 [1]. Similarly, the 2016 Nepal Demographic Health Survey shows a decrease in neonatal mortality rate from 33 deaths in 2011 to 21 deaths in 2016 per 1000 livebirths, accounting for about 65% and 53% of infant and under-five mortality, respectively [2]. Large inequities in accessing and utilizing maternal and newborn services remain between rural and urban populations. Examples include proportion delivered in a health facility (rural 44% vs. urban 69%), four or more antenatal care (ANC) visits (rural 62% vs. urban 75%), and postnatal care within the first 2 days after birth (rural 48% vs. urban 64%) [2].

There are many real and perceived barriers to accessing care, particularly for women in rural areas of low-income countries. These delays are often characterized using Thaddeus and Maine’s “three delays” model: (1) deciding to seek care, (2) reaching a facility, and (3) receiving quality care upon arrival [3]. While initially used to elucidate barriers to improved maternal health and survival, the model can be extended to address access to care and care-seeking practices for newborns with danger signs [4–6].

Nepal’s government has aimed to improve maternal and newborn health outcomes through addressing supply and demand-side barriers to service uptake. The National Safe Motherhood Program (NSMP) [7, 8] aims to provide free 24/7 delivery care, financial incentives for accessing antenatal care (ANC), and delivery services in a facility, birth preparedness, and payments to health facilities to cover free care [8]. The Community-Based Integrated Management of Neonatal and Childhood Illnesses (CB-IMNCI) [9] relies on female community health volunteers (FCHVs) and health workers at health posts to provide counseling for maternal, newborn, and child health to dispense essential commodities and refer upon identification of danger signs [9]. The Effectiveness of these efforts depends on timely recognition of danger signs followed by immediate care-seeking.

Studies in Nepal assessing barriers to skilled ANC and facility delivery were conducted prior to implementation of NSMP and CB-IMNCI. Few studies, however, focus on care-seeking for maternal and newborn illness [10–15]. Reasons for sub-optimal utilization of free maternal and newborn services in facilities in rural Nepal remain unclear, despite the abovementioned efforts to remove financial barriers and link community volunteers to these primary health care facilities. We characterized the processes and factors behind recognition of perceived maternal or newborn complications, the decision-making process, and care-seeking behavior among families and communities who experienced a maternal complication or neonatal illness, some of which resulted in death, in a rural setting of Nepal.

## Methods

### Study site

Bordering India, Sarlahi District (population ~ 750,000) in the southern plains of Nepal is divided into 96 Village Development Committees (VDCs) and 6 municipalities [16]. Public facilities include 1 district hospital (with an OB/GYN doctor position) and 21 birthing centers at the primary level; additionally, there are 2 private facilities (1 Family Planning Association of Nepal Clinic and the other a community hospital) that are supposed to provide 24/7 delivery care staffed by nurses and/or auxiliary nurse midwives (ANMs). In our study area, about 42% of pregnant women deliver at a health facility (data not published). When reporting results, the term facility is used generally and encompasses all types of facilities, unless otherwise specified.

### Study design

Our study was complementary to a multi-country study funded by USAID through the Translating Research into Action Project (TRAction). Details on conceptual framework, research questions, instruments, and analysis can be found in a separate paper [17]. This study collected data on 32 event narratives: 6 maternal death, 10 maternal illness, 6 neonatal death, and 10 neonatal illness cases, in line with a common multi-country protocol of having a minimum of 5 cases per case type to have enough cases to reach saturation [17]. Despite the fact that maternal deaths are a rare event in our study area, we were able to collect 6 maternal and newborn death cases each. For each event narrative, a small group interview was conducted with the primary caregiver for the ill focal woman or newborn and a group of 2–4 other witnesses, who were other family or community members (including care providers) present during the onset of the illness and/or the process that followed. The small group interviews included the witnesses in order to maximize information for the event narratives. For the 10 maternal illness cases, a separate in-depth interview (IDI) was first conducted with the focal woman experiencing the complication, which was then followed by the small group interviews in order to gain personal insight on the focal woman’s experience without the influence of other family members, which may happen in a group setting in this study area. Thus, 10 IDIs and 32 small group interviews were conducted for the 32 illness narratives (Table 1).

**Table 1:** Summary of types of illness event narratives and number of interviews

Type of event narratives	Target group	Methodology	No. of interviews
Maternal death	Main caregiver and 2–4 witnesses present	Illness narrative: small group interview only	6
Maternal illness	Focal woman and 2–4 witnesses present	Illness narrative: in-depth interview + small group interview	20
Neonatal death	Main caregiver and 2–4 witnesses present	Illness narrative: small group interview only	6
Neonatal illness	Main caregiver and 2–4 witnesses present	Illness narrative: small group interview only	10
TOTAL			42

This narrative development activity was nested within a large cluster-randomized community-based trial of the impact of topical applications of sunflower seed oil (compared to mustard oil) to newborn babies on neonatal mortality and morbidity in rural Sarlahi District, Nepal (NCT01177111). The parent trial was implemented in a subset of the district (34 VDCs) by the Nepal Nutrition Intervention Project—Sarlahi (NNIPS), Johns Hopkins Bloomberg School of Public Health, and Nepal Netra Jyoti Sangh; activities included enrolling approximately 30,000 pregnant women and following them and their newborn babies. Post-delivery home visits by field workers on days 1, 3, 7, 10, 14, 21, and 28 allowed for collection of data on labor and delivery circumstances and maternal and neonatal health conditions through the first month of life.

In the event of deaths among pregnant or recently delivered women or newborns, verbal autopsies were routinely conducted in the parent trial. Both the maternal and newborn verbal autopsies are conducted by trained field supervisors upon notification of a death. The field supervisors approach the family members at the earliest possible time (after an appropriate mourning period) to record information on the following: date and approximate time of death, the morbidities present or observed before/during the time of death, care-seeking patterns, and open description regarding the process of death of the baby or woman.

### Sampling method

We utilized the data collected in the larger parent trial to select illness and death events for the 32 event narratives using criterion sampling strategy. Inclusion criteria for maternal cases included the following: female, married, age 15–49 years, parent trial participant, residing in study area, gave birth in the previous 6 months, and quantitative data from the parent trial indicated maternal complications during late pregnancy (third trimester), delivery, or postpartum period. These complications (defined below) were identified prospectively from self-reported maternal morbidity from interviews with the focal woman conducted during the monthly pregnancy visits, immediate postpartum visit, and after the first week postpartum visit in the parent trial. The complications were categorized into the following four groups of the most common complications: (1) postpartum hemorrhage (PPH): the woman reported excessive bleeding at the time of birth or soon after, (2) eclampsia: the woman reported convulsions excluding epileptic fit in the absence of high fever during pregnancy or intrapartum period, (3) puerperal sepsis: the woman reported fever along with foul-smelling vaginal discharge or lower abdominal pain, and (4) prolonged labor: the woman reported labor pains lasted > 24 h. All cases are based on self-reported signs in the absence of medical diagnosis and are thus considered suspected cases of PPH, pre/eclampsia, puerperal sepsis, or prolonged/obstructed labor. These definitions were used in previous studies of self-reported morbidities in South Asia based on WHO Integrated Management of Pregnancy and Childbirth (IMPAC) guidelines [18, 19].

Unlike the other TRAction multi-country studies, we did not exclude women who gave birth at a facility and developed the complication or died prior to discharge and did not limit the maternal complications to only perceived PPH cases. We purposively selected the majority of the maternal complication cases to be perceived PPH cases but also selected a small number of possible eclampsia, sepsis, and prolonged labor cases to see if there were differences in recognition, decision-making, and care-seeking pattern by type of complications. The small number of non-PPH complications is not representative of the cause distribution of maternal complications in the study area. Maternal verbal autopsies, routinely collected as part of the parent trial, were used to identify maternal death cases, defined as a death during pregnancy or within 42 days of cessation of pregnancy from any cause related to the pregnancy or its management; we additionally required that the death occurred within 6 months prior to the family interview.

The inclusion criteria for newborn cases included that they be born in the last 6 months, enrolled in the parent trial, and met our definition of neonatal illness, based on data collected during the 7 home visits conducted in the first 28 days of life (routine parent trial data collection visits). For the purpose of this study, newborn illness was defined as one or more of the following signs: fever, convulsion, difficulty breathing, feeding problems, and skin feels cold. Newborn verbal autopsies were reviewed to identify newborn deaths resulting from an illness event with any one of the above mentioned signs.

### Data collection

The research questions, instrument details, and data collection process are reported in a separate paper [17]. A team of six female interviewers with prior qualitative data collection training and experience were trained for a month. The interviews were completed for all cases between February and October, 2016, and were conducted in pairs by a trained interviewer and note-taker predominantly in Maithili (the local language). Ongoing supervision and assistance for quality assurance and periodic re-training were conducted by the first author. The interviews were audio recorded, transcribed verbatim into Nepali using the notes and audio recordings by the interviewers, and then translated into English for analysis.

### Analysis

The quality of the transcribed notes and entire translations were cross-checked for accuracy by a Maithili-speaking employee and the first author, respectively. Atlas Ti software was used for coding and analysis. Each transcript was read through several times; passages were highlighted and coded for content analysis using a standard codebook developed a priori. Matrices for each case were developed along the three research domains (illness recognition, decision-making, and care-seeking) and common themes were identified corresponding to each domain. Any text that could not be categorized with the initial coding scheme was given a new code. Comparisons were made within and between the four case groups. The results are presented according to the main domains and comparisons made between and within the cases. When referencing care-seeking, we use specific terms to describe types of providers; these terms are described in Table 2.

**Table 2:** Description of health providers, by type of provider, training and type of care provided, and location of care; Sarlahi District, Nepal

Provider	Training and type of care provided	Location
Traditional/informal provider		
Traditional birth attendant	May have some training in clean delivery and newborn health Attends home-based births	Patient’s home
Traditional healer	Shamans referred to as “dhami/jhakri” Provides healing through spiritual cleansing for illnesses believed to be caused by spirit possession	Patient’s home Healer’s home
Village doctors	Village doctors or pharmacy doctors who reside nearby May have some training (e.g., health assistant—3 academic years or community medical auxiliary—2 academic years) but often informal and based on experience Some have their own pharmacy shop Provides allopathic medicines, injections (including uterotonics), and IV solutions	Patient’s home Doctor’s home/pharmacy shop
Skilled/formal provider		
Doctor	Certified as Bachelor of Medicine and Bachelor of Surgery (MBBS) doctor^1^	Clinic/hospital
Nurse	Certified as nurse	Clinic/hospital/birthing centers
Auxiliary nurse midwife	Certified as auxiliary nurse/midwife^2^	Clinic/hospital/birthing centers

^1^Six years of Bachelor’s degree level training

^2^Eighteen months training after high school

### Ethical approval

Written informed consent was obtained from all the respondents. The Johns Hopkins Bloomberg School of Public Health Institutional Review Board (Baltimore, USA) and the Nepal Health Research Council, Ministry of Health and Population (Kathmandu, Nepal) reviewed and approved this study prior to the start of fieldwork.

## Results

### Background characteristics

A total of 101 people participated in the 32 small group interviews (excluding the focal women in the IDI) of which 14 were men who were family members who witnessed the illness event. Ten focal women participated in IDIs among which 9 also participated in the small group interviews for the maternal illness event narratives. The median age of maternal cases was 20 years and ranged from 16 to 40 years. Four of the 6 newborn death cases occurred within 3 days after birth. The majority of the deliveries were at home for newborn cases and at a health facility for the maternal cases. A description of health providers, their training, and the location of care provided is included in Table 2. In addition, a summary of pregnancy profile, illness signs, and outcome for each illness narrative case is presented (see Additional file 1).

### A. Maternal death and complication cases

#### Illness recognition

Among the six maternal death cases, headaches and excessive bleeding were reported in three cases each. Two of the maternal death cases from eclampsia also had signs of swelling of the body, headache, vomiting, convulsions, and stiffness of the limbs. One pre-eclampsia case had reported of headache, swollen body, vomiting, unconsciousness, and high blood pressure upon measurement. Other recognized signs were rolling of the eyes, fever, and signs of cold. Of the ten maternal complication cases, excessive bleeding, unconsciousness, and convulsions (one during the final month of pregnancy and the other postpartum) were reported. One case had both excessive bleeding and convulsions occur soon after delivery.

In the three PPH maternal death cases, the family members or health worker present at the time recognized the bleeding. In the eclampsia and pre-eclampsia cases, the headaches were initially recognized by the focal woman herself and the signs of vomiting, convulsions, and loss of consciousness were recognized by the mother-in-law or mother. In most cases, the focal woman who had the problem informed the female members of the family first of their problem.

Among the various signs, excessive bleeding was very subjective and recognizing this as a severe problem by the focal woman and other witnesses was delayed due to factors such as lack of prior experience (especially in primigravida cases) or the perception that bleeding was normal (i.e., by female members of the family or health workers). For example, the sister-in-law of a woman with PPH said “This new mother (focal woman’s name), had the same amount of bleeding that we had when we had our babies. She had just like us. That is why I said it was all right” (MC-1). Prior pregnancy was also reported as an enabler for recognizing the severity of PPH sign. One focal woman stated “You know the bleeding should stop after a while, but it didn’t. I have two other children and before this pregnancy, I did not have such an experience” (MC-4).

The focal woman and witnesses to the PPH described the excessive bleeding as severe if bleeding did not decrease, flow was heavy, or bleeding was accompanied by loss of consciousness. One of the traditional birth attendants (TBAs) who did not think the focal woman’s excessive bleeding was severe stated “If the person had fainted or if the person felt nauseous after excessive bleeding then we would consider it as a problem” (MC-5).

In all four of the eclampsia cases, the sign of headache, vomiting, and swelling of the body were not recognized as severe signs until the onset of convulsions and loss of consciousness. Knowledge about other women’s fatal outcomes after similar signs also prompted recognition of the severity of the signs. One of the focal women who had excessive bleeding and convulsions said “In that village of ours, one of the women had the same kind of problem and she died” (MC-9). A sudden inability to conduct household chores helped recognize the problem in other instances. For example, in the prolonged labor case, the focal woman’s brother-in-law reported “When it became difficult for her to work and when she had difficulty in walking about she started to cry and walk up and down. Then only did we know that she was having problems. She did not tell anybody” (MC-3). All of the focal women perceived their problem to be severe, and many of them said they felt like they would die.

Cause attribution for the complications varied. For the eclampsia and pre-eclampsia cases, the initial cause for the signs was believed to be possession by evil spirit or witchcraft (“boksi laageko”), mainly due to the sudden onset of convulsion and/or unconsciousness and the circumstances surrounding it. The prolonged labor was thought to be caused due to weakness while sepsis was thought to be caused by the delivery of the baby. Most of the PPH cases were thought to be due to the delivery process.

#### Decision to seek care

Decision-makers in first seeking care for maternal cases (both survivors and deaths) were usually the mother-in-law or mother, followed by the husband; less commonly, a sister-in-law or neighbor initiated decision-making. As bleeding was often considered normal, the decision to go to a facility was often delayed in favor of a wait-and-see approach as seen in the three home birth PPH cases (MC-1, MC-7, and MC-9). In one case, even when the bleeding started while at the hospital, the mother-in-law did not perceive the bleeding to be excessive and thus informed the nurse only after 7 h without a decrease in bleeding (MC-10). As eclampsia or pre-eclampsia were attributed to “boksi laageko,” traditional healers were called first in all cases; in one case, the traditional healer’s immediate advice prompted the family to seek facility care.

Factors prompting care-seeking outside the home included recognizing signs as severe, proximity to facility (any type of health facility), arrangement and availability of transport (ambulance or private vehicle), availability of money, facility workers being relatives or known acquaintances, referral, and prior use of services; seeking care inside the home from informal providers was enabled by familiarity (prior use) of such services and close proximity (ease of contact). Barriers to seeking care outside of the home included difficulty accessing transport, fuel shortages, restriction on vehicular movement due to local or national strikes (“bandhs”), perceived lack of medicines/supplies in health facility, and illness occurring at night. In many cases, more than one care provider was sought and the main delay in deciding to seek subsequent care was late referral by the previous care provider (five cases) or false reassurances, given by the initial provider, that everything would be fine. “The doctor did the checkup and said that she has convulsion illness which will not go away this soon and will take time. The doctor also said that it will take her 3 days to recover. The doctor told us not to worry and said that he would save both her and her baby’s life. But when it was 5 o clock, she passed away.”—father of focal woman (MD-3).

#### Actual care-seeking pattern

##### Deaths

**Fig. 1:**
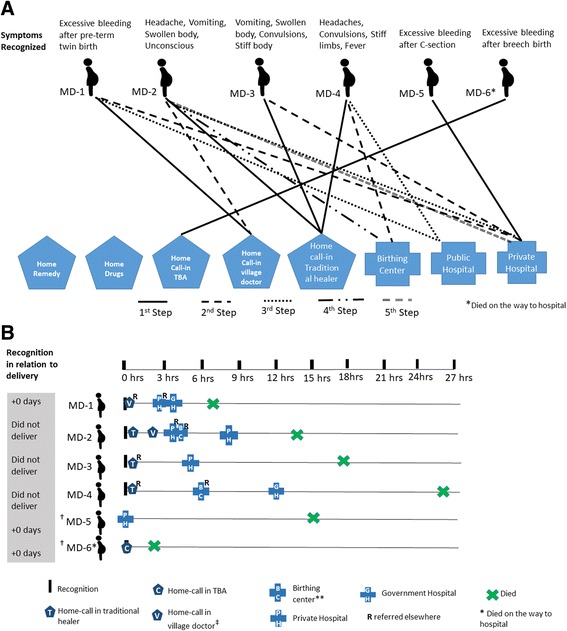
Maternal death cases. **a** Care-seeking steps and **b** care-seeking timing and locations. ‡Village doctor is an informal “doctor” who provides allopathic medicine with little or no training and conducts home visits or runs a pharmacy where care is also provided. **Birthing center is attached to a government primary care facility (health post or primary health care center), staffed by nurse and/or auxiliary nurse midwives that provides free ANC and 24/7 labor/delivery and immediate postpartum care ^†^MD-5 was at a private hospital during onset of symptoms; MD-6, a TBA was present at home to help with the home birth, and a local doctor had been called to give injection to induce labor but had left before the delivery of baby and symptoms recognized

In four of the six maternal death cases, home care from informal providers such as a traditional healer (pre-eclampsia and eclampsia cases) or village doctor was first sought (care-seeking trajectories and timings shown in Fig. 1a, b). Subsequently, all four were first referred to a private hospital or a government health facility, and in three cases (MD-1, MD-2, MD-4), this was followed by referral to a higher level facility. One of the deceased women with PPH was at a private hospital before problem onset and did not seek care elsewhere (MD-5). The other PPH-associated death occurred within 3 hours of homebirth (wherein obstructed labor and breech birth were reported); in this case, rapid progression of illness and distance to health facility were cited as reasons for not seeking care (MD-6) (Fig. 1b). All six maternal deaths occurred within 26 h of recognition (Fig. 1b).

##### Complications

Among four of the seven PPH cases, sign onset and initial care occurred while at a health facility; in two such cases, the woman was discharged despite continuous bleeding, and subsequent care was then sought from a village doctor (MC-10) or another private hospital (MC-8). In one of these facility PPH cases (MC-4), despite a referral to the next level, financial constraints led the mother-in-law to instead summon to the birthing center a village doctor, who gave an injection to stop the bleeding; the woman and relatives then returned home. In three PPH cases where the woman had delivered at home, two sought care from informal providers (traditional healer and/or village doctor) and one did not seek care despite heavy bleeding for 7 days. In two cases of eclampsia (MC-2 and MC-9), a traditional healer was called in first, who then referred care outside of home to a health facility. For the case of prolonged labor (MC-3) and sepsis (MC-6), care was first sought at home from a TBA. Two sources of care were sought in majority of cases, due to direct referral from the first provider or because of continuing signs.

**Fig. 2:**
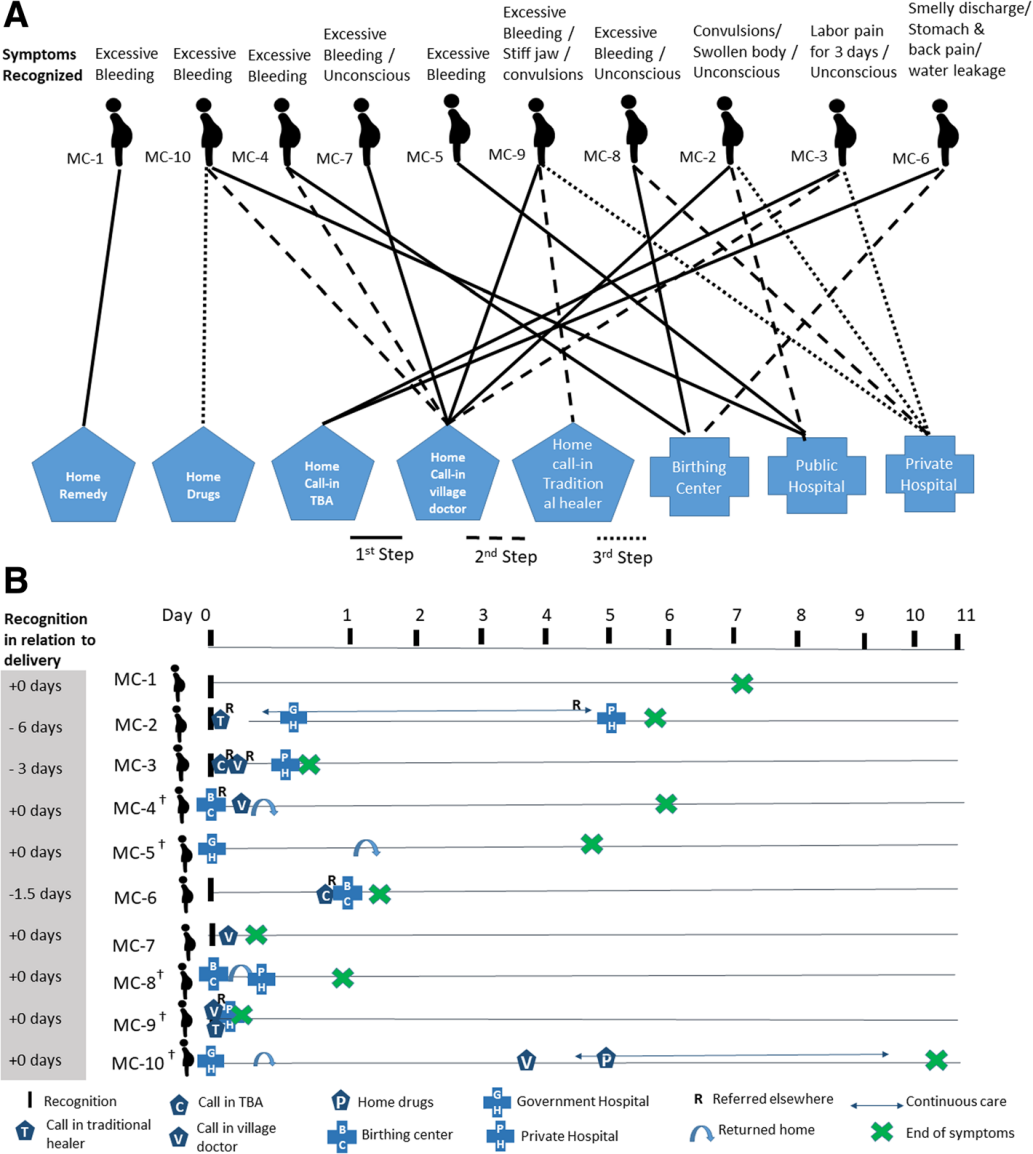
Maternal complication cases. **a** Care-seeking steps and **b** care-seeking timing and location. ^†^MC-4, MC-5, MC-8, and MC-10 were already at a health facility during onset of symptoms; MC-9, a village doctor was present at home to check on the newborn baby before onset of symptoms

The sequence, locations, and timing of care-seeking for the maternal complications are illustrated in Fig. 2a, b. Four of the PPH cases occurred after delivery at a heath facility prior to being discharged (MC-4, MC-5, MC-8, and MC-10), among which, in three cases, they were discharged without any referral despite the woman having excessive bleeding (Fig. 2b). In three other cases (MC-2, MC-3, and MC-9), some form of care was sought at home within an hour of sign recognition. In four cases, the signs were reported as resolved within 24 h of recognition, but in one case, it took 11 days (Fig. 2b).

#### Perceived quality of care

The respondents’ perception of quality of care and satisfaction varied. Positive responses were reported when the focal woman’s condition improved. However, some respondents also noted satisfaction with care in health facilities when signs did not resolve; in these cases the respondent highlighted the fact that health workers provided immediate care, were polite, and did all they could within their capacity before referring elsewhere. Other respondents expressed dissatisfaction with the care provided because the focal woman either died or did not improve after treatment. One of the PPH-related maternal deaths was thought to be due to the negligence by the doctors as the baby was “pulled out” (MD-1). In another instance where the woman died (MD-5), timely delivery of appropriate emergency obstetric care for excessive bleeding following a C-section was delayed due to a shortage of blood for transfusion; a family member traveled to a neighboring district to get the required quantity, but the woman’s condition had worsened before his return.

“The doctor should stay at the hospital at all times, and tell the relatives of the patient ahead of time that your patient has low blood and that the hospital is low on some other supplies, only then can we prepare for such things … Only after she (focal woman) was in a very serious state, the doctor told us to quickly get blood. So at that time, where can we get blood immediately?”—mother in-law (MD-5) discussing the care provided at private hospital.

In a few cases, health workers in both the private and public health facilities were reported as speaking rudely and harshly when the family asked many questions. “I did not like the behavior of the employees there because they used to speak very harshly. The sister (nurse) of the hospital also used to speak very unkindly.”—mother-in-law (MD-2) discussing the birthing center nurse.

A shortage of drugs and supplies at both private and government facilities was a barrier to adequate care resulting in referral and further delay. It was also commonly reported that the family had to buy the medicines and supplies for care sought at government health facilities of all levels (seven cases). “We took her to the hospital. We bought everything and did her treatment. We even had to buy gloves, syringe and everything else. What services did we get even if the government hospital was nearby?”—mother-in-law (MC-4) discussing care sought at birthing center.

### B. Newborn death and illness cases

#### Illness recognition

Of the six newborn death cases, multiple signs were reported for each child. The most common of these were weakness/lethargy (four cases), difficulty breathing (three cases), “cold-like” signs, i.e., upper respiratory tract signs (three cases), and excessive crying (three cases). Fever, not breathing after birth, inability to cry, not drinking milk, cold to touch, convulsions, stiff body and eyes rolled over, abnormal skin color, distended stomach, and deflated stomach were some of the less common signs reported. Signs were usually first recognized by either the grandmother and/or the mother. In two cases where sign onset followed soon after birth, the attending village doctor recognized the signs along with family members. Among three newborn death cases, the problem occurred immediately after birth; two of these were deaths of a twin in which there was no a priori knowledge of a multiple birth (ND-3: preterm home birth and ND-6: term facility birth) (see Additional file 1).

Among the newborn illness cases, the most common reported signs were difficulty breathing, fever, cough, and “cold-like” signs. Difficulty feeding, cold to touch, not breathing at birth, abnormal skin color, distended stomach, convulsions, limp body, and jaundice were less commonly reported. Signs were first recognized by the immediate family members such as the mother, grandmother, and/or aunt (mother’s sister-in-law). In three separate cases, the TBA, a neighbor present during delivery, and the father of the baby also recognized the signs.

In all except one (ND-6) of the newborn deaths, signs were recognized as very severe. “I felt that it was less severe and that it would be fine at home itself. I said that the baby would be well here (home).”—father of newborn (ND-6). Even in a few of the newborn illnesses, the baby’s signs were perceived as severe and chances of survival considered bleak. Weakness, breathing difficulty, and convulsion were mainly perceived as severe signs.

Respondents attributed the neonatal deaths to pneumonia (ND-2, ND-5), preterm birth (ND-3), excessive medication and inadequate water consumption during pregnancy (ND-1), weak baby (ND-4), and cold weather (ND-6). Among all seven surviving illness cases where onset was within 24 h of birth, respondents attributed the cause to pregnancy-related maternal diet (e.g., eating certain foods traditionally classified as “cold” food associated with causing cold (such as sugarcane, yogurt) or lacking nutritious food), pregnancy-related behavior (e.g., taking cold bath), or the cold weather at the time of birth. For instance, the grand aunt of the child born in mid-January (cold season) said “Many people say that the children these days already have pneumonia inside the mother’s womb” (NC-6).

#### Decision to seek care

Among the newborn death cases, care-seeking decision-makers varied; the grandmother or entire family were involved in two cases each. Similarly, the newborn illness cases also saw the entire family or other female members in the household (grandmother, mother, aunt) making the decisions. In one case, the father made the decision and another case it was the TBA.

In all six newborn death and nine illness cases, ultimately, a decision to seek care outside of the home was made, often enabled by readiness/availability of transport and financial resources, prior experience with a provider, or proximity to care. Frequently, a village doctor’s advice was sought prior to going to a health facility, given they usually lived nearby (i.e., within same village), and prior good experience(s) led to expressed sentiment of “trustworthiness.” Prior experience was given as a reason for the decision for choosing a provider as reported by aunt of one child (NC-4) “That doctor [village doctor] is very nice, whatever he gives the children, medicine or injection, the children become well, and he gives good medicines.”

A prompt decision to seek care, however, was compromised by numerous barriers, including a delay in arrangement of transportation and money, family members not considering the signs to be severe, and family circumstances (not able to leave immediately due to another death in the family or needed to take care of home/livestock). Similarly, among the newborn illnesses, commonly reported barriers included difficulty finding transport due to strike and timing of sign recognition being late at night. In only one case was the “chhatiyar” tradition (confinement of mother and newborn until naming and purification ceremony, typically on the sixth day [15, 20, 21]) a barrier to seeking care; in this case (NC-4), the illness was recognized within the first 24 h after birth and was considered serious. When asked why the care was sought only after 6 days, the mother-in-law said “How would I take the recently delivered mother at that time, we shouldn’t”, followed by the sister-in-law “We didn’t take at that time, but after the ‘*chhathiyar*’ we went to village doctor” (NC-4).

#### Actual care-seeking pattern

**Fig. 3:**
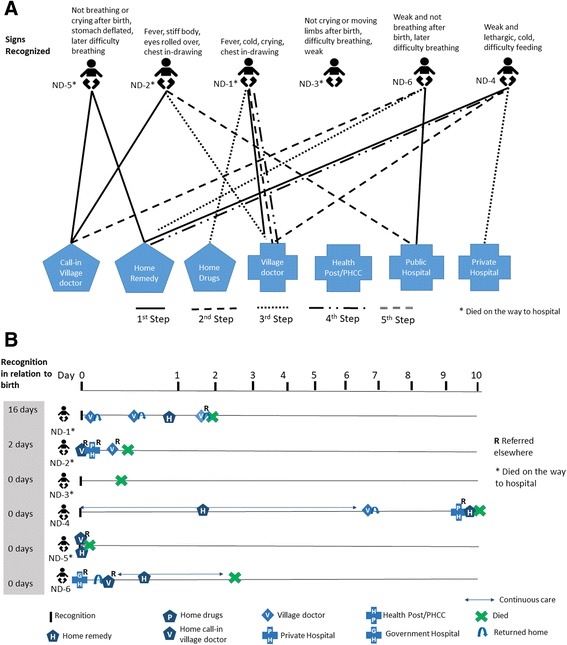
Newborn death cases. **a** Care-seeking steps and **b** care-seeking timing and location

Newborn death care-seeking steps and timeline are illustrated in Fig. 3a, b, respectively. In contrast to the maternal death cases, a caretaker commonly turned to home remedies, including massage with oil heated with garlic, mouth-to-mouth resuscitation, massaging of placenta, steam treatment with “vicks,” and warming the baby near a fire lit from the roots of “lahari daal” plant (a type of lentil plant) understood to have healing properties. Seeking advice from village doctors was common; in one case (ND-1), three different village doctors were consulted, and the baby died without any care sought from formal providers. In general, late decision-making with regards to formal providers was common; in four cases, death occurred on the way to the facility (Fig. 3b). Even with a prompt decision to seek care in the case of home-delivered preterm twins (ND-3), 5 h passed while arrangements were made for money and an ambulance, resulting in death of the second twin. Four of the newborns died within 24 h of first recognition of signs, and one died within an hour.

**Fig. 4:**
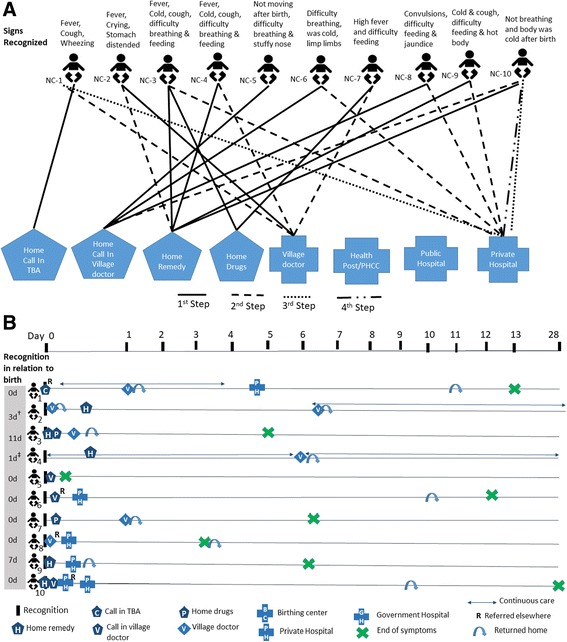
Newborn complication cases. **a** Care-seeking steps and **b** care-seeking timing and location. †Illness not resolved yet; still on continuous care from same local doctor every week. Not resolved even after 24 weeks since the onset of signs (NC-2). ‡Illness not resolved yet; still on continuous care from same local doctor every 11th day. Some signs not resolved yet even after 15 weeks since onset of signs (NC-4)

Some level of care was sought in all cases of neonatal illness (Fig. 4a, b). Home remedies or bringing medicines home were predominately the first choice, followed by a subsequent step where local providers (village doctors and/or TBA) were sought, sometimes repeatedly despite no improvement (NC-2, NC-4) (Fig. 4b). Care was eventually sought from facilities for only half the cases, and in all these instances, these were private providers. Among the few cases where the baby was hospitalized, time of stay ranged from 3 to 9 days.

#### Perceived quality of care

Similar to the maternal cases, respondents largely indicated satisfaction with the care sought for babies’ illnesses, as signs resolved or improved. One family praised the accommodating nature of a private hospital where their bill was reduced from Rs. 50,000 (~ US$ 500) to Rs. 10,000 (~ US$ 100) since they were poor. Despite fatal outcomes, numerous respondents reported satisfaction with care provided, noting that signs of improvement were initially made (ND-1 and ND-4) or health workers tried their best but ultimately had to refer elsewhere (ND-5 and ND-6). “The doctor said he would do something so that the baby survives for two or three hours and told me to take the baby and go to Sitamadi (nearest large city in India)”—father of baby (ND-6) about being referred from the district hospital (located near the Indian border).

In all the newborn cases, health workers were characterized by respondents as “polite” and/or “good.” Only once did a respondent comment on shortage of supplies: lack of oxygen was given as the reason for referral by one of the health facilities (ND-4).

## Discussion

We characterized illness recognition, decision-making and care-seeking behavior for fatal and non-fatal cases of maternal and newborn illnesses in a rural community in Nepal, following implementation of the government’s recent large-scale programs, NSMP and Community-Based Newborn Care Program (CB-NCP), which was implemented prior to its replacement by the CB-IMNCI. To do so, we adopted Thaddeus and Maine’s three delays model to explore the delays of care-seeking for maternal and newborn illnesses.

Recognition that the illness was severe was a requisite to prompt decision-making related to initial care-seeking. There was some delay in the decision to seek care in a few maternal and newborn cases but for different reasons. For the maternal cases, the severity of some PPH cases was recognized late, only after worsening of signs or loss of consciousness, as senior female family members or TBAs did not consider the bleeding to be severe; this perception was often based on their own past experiences, and bleeding was considered normal, resulting in a wait-and-see approach, as observed in several other studies [14, 22, 23]. For the newborn cases, delays in the decision to seek care were due to the use of home remedies or drugs brought from a pharmacy (in these cases, it was unclear whether or not a pharmacist offered advice), timing of illness event at night, lack of perceived severity, no one to take the baby to a facility, and the tradition of “chhatiyar.” This cultural norm of a 6-day postpartum confinement for newborns and mothers (“chhatiyar”) has been previously reported as an important barrier to seeking prompt care in Nepal and South Asia [15, 24, 25].

Spiritual causes were commonly reported for eclampsia and pre-eclampsia maternal cases, resulting in a traditional healer being called first, similar to other studies in the region [15, 22, 24]. Only when spiritual causes were ruled out or treatment failed was the mother or newborn referred or the family decided to seek care at a health facility, thus delaying decision to seek appropriate care (delay 1). This may be because the traditional belief that expectant mothers and newborns are susceptible to evil spirits is still deeply rooted in the culture, especially in rural areas [11, 20, 22]. In contrast, there was no care sought from traditional healers for any of the newborn cases unlike other studies in Nepal [15, 24].

There were many similarities as well as differences in care-seeking practice between the maternal and newborn cases. The first care was sought at home from TBAs, traditional healers, or village doctors in all of the maternal cases when the illness onset occurred at home, consistent with other studies [15, 24, 26, 27]. In our study district, past research shows that TBA and village doctors are commonly summoned to attend homebirths, with the latter often being requested to provide injections such as oxytocin or other uterotonics [28]. Among the newborn cases, the first care sought besides home remedies was from a village doctor called to the home or at their pharmacy/clinic. Care-seeking from informal providers for newborn illness is common in South Asia [24, 29–31]; as we also observed, this is often due to such providers being easily accessible, working flexible hours, and high levels of trust and familiarity [22, 32, 33].

Evidence of over-reliance on private informal providers such as village doctors (for both maternal and newborn cases) and TBA and traditional healers (maternal cases) highlights the demand and supply gap in the health care sector. Timely referral by informal providers played an important role in facilitating swift decisions to seek skilled care at a facility for maternal death. There were more instances of caretakers of sick newborns not complying with referral advice among the newborn deaths than maternal cases. The lack of care or advice for care-seeking sought from FCHVs who are supposed to be the frontline health workers in the community reflects the need to increase the demand for their services within the study community. Eventually, facility-based care was sought for cases of maternal complications; when multiple facilities were consulted, the public facility was normally the initial step, possibly due to increased awareness and availability of free services provided at birthing centers under NSMP. In contrast, for newborn illnesses, government facilities were more likely to be bypassed, in favor of private facilities, perhaps suggesting that community members perceive limited capability of public facilities to provide specialized care for newborn illnesses [32].

In summary, among the maternal deaths, ineffective decision-making, resulting in initially seeking care from informal providers, delayed timely skilled care (delay 1) and a subsequent delay in receiving appropriate care (delay 3) when arriving at the facility, as observed elsewhere [22, 34]. For newborn deaths, the first delay appeared most critical; the decision to seek skilled care or to act upon referral advice was frequently delayed and made only after failed home remedies or unsuccessful attempts to resolve the problem through consults with village doctors; in numerous cases, these delays likely contributed directly to the death. These findings are consistent with other systematic reviews [35] and studies on care-seeking for neonatal illness in Nepal and elsewhere [5, 15, 36].

This study has several strengths, including nesting activities within a prospective study allowing for rapid community-based identification of cases, including both fatal and non-fatal cases for both mothers and newborns and a mix of maternal complications beyond PPH, which is often the sole focus of other efforts. However, our work was restricted to one district in southern Nepal, and care-seeking patterns across districts or across regions of the country may differ. Further, we relied on self-reports, and recall may have been biased by time since event or severity of the cases; we tried to mitigate this by restricting recall period length to 6 months and using small group interviews to maximize information and triangulation while constructing the narratives. This study might have been strengthened by the inclusion of the health workers in separate interviews to triangulate some of the findings.

## Conclusion

Ongoing programs need to strengthen demand for skilled care and improve access and quality of newborn and maternal services. Obstacles to care-seeking in our study area included not recognizing and understanding the severity of danger signs, reliance on wait-and-see approaches, and a preference to first treat the illness by informal providers in the community. When antenatal or postnatal counseling emphasizes recognition of illnesses and provision of referrals by community health workers, care-seeking for both maternal and newborn illnesses can improve significantly [37]. Combining such counseling along with community visits by FCHVs is currently mandated within the NSMP and is integrated into the neonatal component within the CB-IMCI program, but more intensive efforts are needed to educate communities to recognize pregnancy, intrapartum, and newborn danger signs and prepare (money, transport, identify health facility, etc.) for care-seeking from skilled providers upon recognizing the severity of danger signs.

One promising demand-side model that might be further explored is the implementation of Pregnant Women’s Groups (PWG) or mothers’ groups at the community level [38]. The international non-governmental organization PLAN Nepal is currently implementing these groups in 15 districts, including Sarlahi. Such groups, often facilitated by FCHVs, are comprised of 8–15 pregnant women and postnatal mothers who meet monthly to share essential health information related to pregnancy, birth, and newborn care [38]. Additional engagement of key decision-makers (i.e., husbands, senior female family members) in such PWGs might prove effective in this setting. Although husbands and in-laws are encouraged to participate in the PWGs as part of PLAN Nepal’s implementation, their role in maternal and newborn health care-seeking may be strengthened by piloting further activities. One such example might be an initiative where selected married men are actively engaged in maternal and newborn health care, such as couple-based ANC and/or PNC visits, and then encouraged to inform their peers about their experiences, promoting wider participation [39]. Alternatively, another approach might include community sensitization at the VDC level through training and mobilization of safe motherhood promoters [40]. These female promoters could complement ongoing FCHV efforts, by disseminating maternal and newborn health information at the community level, to address gaps in existing programs.

On the supply side, while the NSMP has resulted in greatly increased accessibility to delivery care at 24/7 birthing centers and hospitals, primary health care facilities are open only briefly (4–5 h) during work days. This reduced accessibility, combined with lack of awareness of services, played a major role in respondents seeking care from private facilities for newborn illnesses.

The private sectors’ (both formal and informal providers) increasing role in providing newborn care services in rural settings needs to be studied further and interventions piloted in engaging this sector to provide quality care where service gaps exists. Engaging the private informal practitioners and training them to recognize pregnancy/delivery and neonatal danger signs and to promptly refer to appropriate health facilities have been shown to reduce maternal and perinatal deaths [33, 41–43]. The effectiveness, feasibility, and scalability of such an approach can be piloted, and further research on including this private sector especially in newborn care is needed.

## Additional file

Additional file 1: Table S1. Maternal death event narrative summaries. **Table S2.** Maternal illness event narrative summaries. **Table S3.** Newborn death event narrative summaries. **Table S4.** Newborn illness event narrative summaries. The data displays a summary on each individual illness narrative case for each of the 32 cases. The summary includes location of case (VDC), age of focal woman (for maternal death and complication cases), gender of newborn (for newborn death and complication cases), pregnancy profile, illness symptoms/sign, and outcome of the illness event which includes where and when in the case of death and when the illness was resolved (since illness recognition) for the complication cases. (DOCX 41 kb)

## Abbreviations

ANC: Antenatal care
ANM: Auxiliary nurse midwives
CB-IMNCI: Community-Based Integrated Management of Neonatal and Childhood Illnesses
CB-NCP: Community-Based Newborn Care Program
FCHV: Female community health volunteer
IDI: In-depth interview
MC: Maternal complication
MD: Maternal death
NC: Newborn complication
ND: Newborn death
NNIPS: Nepal Nutrition Intervention Project—Sarlahi
NSMP: National Safe Motherhood Program
OB/GYN: Obstetrics/gynecology
PPH: Postpartum hemorrhage
PWG: Pregnant Women’s Group
TBA: Traditional birth attendant
VDC: Village Development Committee
WHO: World Health Organization
