# Supplementary material for: Illness recognition, decision-making, and care-seeking for maternal and newborn complications: a qualitative study in Sarlahi District, Nepal
Source: J Health Popul Nutr. 2017 Dec 21;36(Suppl 1):45. doi: 10.1186/s41043-017-0123-z (PMC5764053; doi:10.1186/s41043-017-0123-z)
Supplement: Additional file 1: Table S1. — Maternal death event narrative summaries. Table S2. Maternal illness event narrative summaries. Table S3. Newborn death event narrative summaries. Table S4. Newborn illness event narrative summaries. The data displays a summary on each individual illness narrative case for each of the 32 cases. The summary includes location of case (VDC), age of focal woman (for maternal death and complication cases), gender of newborn (for newborn death and complication cases), pregnancy profile, illness symptoms/sign, and outcome of the illness event which includes where and when in the case of death and when the illness was resolved (since illness recognition) for the complication cases. (DOCX 41 kb) [file 41043_2017_123_MOESM1_ESM.docx]

**Table S1. Maternal death event narrative summaries**

| **Case type** | **Pregnancy Profile** | **Illness Symptoms** | **Outcome** |
| --- | --- | --- | --- |
| **MD-1**  Kabilasi VDC  20 years old  PPH | First pregnancy, pregnant with twins (not aware ahead of time), and 4 ANC visits done.  During her seventh month she went into labor and day before she had water flowing  One twin (son) born breech at home and died, the second baby birth was stillborn and delivered at district hospital (Malangwa) | Focal woman was screaming after second baby was born and then heavy bleeding started | Died at the hospital 7 hours after symptom recognition |
| **MD-2**  Sundarpur VDC  17 years old  Pre-eclampsia | First pregnancy, 3 ANC visits reported.  Illness event took place when the focal woman was six months pregnant | Focal woman complained of headache and was sleeping.  Later had vomiting, shivering, eyes turning, became unconsciousness and swollen body  High BP (240) reported after getting checked at health facility | Died at the hospital 14 hours after symptom recognition |
| **MD-3**  Pharahadawa VDC  20 years old  Eclampsia | First pregnancy, 3 ANC visits reported  Illness event took place when the focal woman was eight months pregnant | Focal woman suddenly started vomiting, blood in vomit  She then started convulsing and stiffness of body. Also had swelling of her feet were very big | Died at the hospital approximately 18 hours after symptom recognition |
| **MD-4**  Ishwarpur  VDC  16 years old  Eclampsia | First pregnancy, no ANC checkup  Illness event took place when the focal woman was eight months pregnant | Focal woman complained of headache and then started convulsing, limbs became stiff and fever | Died at the hospital approximately 26 hours after symptom recognition |
| **MD-5**  Pipariya VDC  30 years old  PPH | Seven previous pregnancies  (6 daughters and 1 miscarriage)  Gone for three ultrasounds total but no ANC checkup. Facility birth – Janaki Healthcare Research Center (Private)  Prolonged labor and high BP and doctor said if C-section delayed then she may have convulsions so had to do C-section | Excessive bleeding after C-section | Died at the hospital 15 hours after symptom recognition |
| **MD-6**  Mahinathpur  VDC  20 years old  PPH | Two previous pregnancies,  No ANC checkup  Home birth | Breech birth and baby had a large head and thin body. Baby died shortly after and excessive bleeding | Died on the way to hospital in India  Died two hours after symptom recognition |

**Table S2. Maternal illness event narrative summaries**

| **Case type** | **Pregnancy Profile** | **Illness Symptoms** | **Outcome** |
| --- | --- | --- | --- |
| **MC-1**  Sundarpur VDC  16 years old  PPH | First pregnancy, 3 ANC visits,  Home birth | Excessive bleeding soon after delivery for seven days according to the focal woman | Illness resolved around 7 days from symptom recognition |
| **MC-2**  Salempur  VDC  16 years old  Eclampsia | First pregnancy, 6 ANC visits  Facility birth (C-section) – Janakpur Janaki Hospital (Private) | Convulsions in 9^th^ month of pregnancy and later body was swollen | Cesarean section performed on day 5 since symptom recognition and then resolved |
| **MC-3**  Gadaiya  VDC  23 years old  Prolonged labor | Third pregnancy, 4 ANC visits,  Facility birth – Majorganj, India (Private)  Had symptoms of night blindness in last month of pregnancy  Had water & blood discharge 4-5 days prior to baby birth | Labor pains for three days and became unconscious | Illness resolved around 13 hours from symptom recognition |
| **MC-4**  Ishwarpur  VDC  23 years old  PPH | Third pregnancy, 4 ANC visits  Facility birth – Ishwarpur Birthing Center  Ultrasound done | Focal woman had heavy bleeding soon after delivery while at the birthing center  Bleeding continuously and lost consciousness. She was also vomiting and could not open her eyes but could hear. Eyes were sunken in | Illness resolved within 6 days from symptom recognition |
| **MC-5**  Mohanpur  VDC  22 years old  PPH | Second pregnancy, 6 ANC visits,  Facility birth – District hospital (Malangwa) | Heavy bleeding 5 minutes after baby was born in the hospital  Bleeding lasted for four days continuously and she felt weak (discharged from hospital on second day after delivery) | Illness resolved within 5 days from symptom recognition |
| **MC-6**  Hempur VDC  20 years old  Sepsis | First pregnancy, 4 ANC visits  Facility birth – Jamuniya Birthing Center  Had done two ultrasound visits  She reported being 20 days past her due date at the time of delivery | Water discharge a bit smelly in the morning  Stomach pain, water leakage, and back pain | Illness resolved 32 hours since symptom recognition |
| **MC-7**  Manpur VDC  40 years old  PPH | Fifth pregnancy, One ANC visit,  Home birth  Watery discharge for 3-4 days prior to delivery | Heavy bleeding and body was shivering two hours after giving birth, became unconscious | Illness resolved at 7 hours from symptom recognition |
| **MC-8**  Ishwarpur VDC  Pahadi (Hill person)  20 years old  PPH | First pregnancy, 6 ANC visits  Facility birth – Ishwarpur Birthing Center  Ultrasound was done and said baby was breech position | Heavy bleeding, vomiting then became unconscious, eyes were rolled over (only white of eyes seen) half an hour after baby was born | Illness resolved in 24 hours from symptom recognition |
| **MC-9**  Salempur VDC  22 years old  PPH and possible Eclampsia | Third pregnancy, 4 ANC visits,  Home Delivery (Maiti)  Ultrasound also done | Excessive bleeding soon after giving birth and also had stiffness of jaws and then convulsions simultaneously | Illness resolved in 3 hours from symptom recognition |
| **MC-10**  Babargunj VDC  16 years old  PPH | First pregnancy, 6 ANC visits,  Facility birth - Janakpur zonal hospital (Pubic)  Had done ultrasound | Had a lot of bleeding after delivery for several hours and focal woman recognized it as unusual as it was not decreasing and the type of blood was also of concern (pieces of flesh and murky red color) | Illness resolved by 11 days from symptom recognition |

**Table S3. Newborn death event narrative summaries**

| **Case type** | **Pregnancy Profile** | **Illness signs** | **Outcome** |
| --- | --- | --- | --- |
| **ND-1**  Kishanpur VDC  Daughter | First pregnancy; 5 ANC visits  Ultrasound also done twice  Home birth had called local doctor from neighboring VDC who gave injections day before baby was delivered and then TBA came to help. Both called due to problem. | On the 16^th^ day since birth, the baby had fever first and also signs of cold and crying non-stop and chest in-drawing  Baby had distended abdomen and noise coming from abdomen like ‘dhui dhui’ | Newborn died on the way to health facility in about 42 hours of symptom recognition and was 18 days old |
| **ND-2**  Janakinagar  VDC  Daughter | First pregnancy; 5 ANC visit  Ultrasound home visit by NNIPS.  Facility birth – Bharatwa Birthing Center  Baby did not cry immediately after birth and with steam treatment from nearby medical shop and help of BC nurse, it started crying | On day 2 after birth, the baby started making fists tight and body became stiff and eyes were rolled over, and had fever  Fever (102 degrees) and baby crying and also had chest indrawing. | Newborn died on the way to health facility in 10 hours from symptom recognition and was 3 days old |
| **ND-3**  Laxmipur  VDC  Son | Second pregnancy, 4 ANC visits  Ultrasound done and knew about the twins (both sons)  At home labor pain began and delivery was at home at 7 months pregnant (pre-term).  Twins – first baby was fine but died after a month @ 1100 gms  Second baby had problems | Second twin born was not crying, not moving limbs, or drinking milk , eyes closed, and only breathing slightly, weak heartbeat  Baby skin color kept changing from yellow to white then black and tongue was cold | Newborn died on the way to the hospital in about 9 hours from symptom recognition and was less than a day old |
| **ND-4**  Babargunj VDC  Daughter | First pregnancy, 4 ANC visits  Baby girl born at home with help of traditional birth attendant  Baby was thin, weak and lethargic when born | On day 6 since birth, baby was weak and lethargic and had cold symptoms  Not drinking milk on day 12 and same condition as before | Newborn died a home about 10 days from symptom recognition was 16 days old |
| **ND-5**  Kishanpur VDC  Son | Fifth pregnancy (1 son had died)  No ANC visits  Home birth - help of local doctor who was called to give injection. Baby was stuck for a bit and then came out. | Baby was not breathing or crying after birth  Stomach was deflate and like attached to the back  Baby was cold to touch | Newborn died on the way to the hospital about an hour from symptom recognition and was less than a day old |
| **ND-6**  Pipariya  VDC  Son | Fifth pregnancy; 5 ANC visits  No ultrasound done due to bandh but did ultrasound in Malangwa during the time of labor and found out about the twins  Facility birth – district hospital, Malangwa  Twin births  Baby girl (2010 gms) born in Malangwa hospital  Baby bi was born 4 hours after the first twin and had problems | Baby was weak, not breathing for 1-2 hours when born  Then after it started breathing but with difficulty  Baby’s hands and body were quivering like a little convulsions  Body kept shivering, eyes were dull | Newborn died a home about 58 hours from symptom recognition and was less than 3 days old |

**Table S4. Newborn illness event narrative summaries**

| **Case type** | **Pregnancy profile** | **Illness signs** | **Outcome** |
| --- | --- | --- | --- |
| **NC-1**  Bela VDC  Son | First pregnancy,  No ANC visits  Home birthh due to lack of money for transport, no transport of own and no male family members to take them | Baby was not breathing when it was born  Later had pneumonia, fever, wheezing, cough  Becomes sick and then all right again sick and all right | Newborn illness resolved within day 13 from symptom recognition |
| **NC-2**  Barahathawa VDC  Daughter | First pregnancy  4 ANC visits  Labor pains started 7 days before baby was born  Taken to the BC when labor pain started at  Facility birth: Barahathawa BC | Fever, crying differently and stomach distended from day 3 after birth  The same symptoms fever, stomach ache and cough reoccur after 6-7 days | Newborn illness no resolved as the baby is still taken for care and given medicines from the same local doctor every week  *Note: interview was done when baby was almost 6 months old |
| **NC-3**  Salempur  VDC  Son | Sixth pregnancy (1 son died), 2 ANC visits  Home birth | Day 11 since birth had fever, cold and cough, not sucking milk, difficulty breathing | Newborn illness resolved on 5^th^ day from symptom recognition |
| **NC-4**  Barahathawa VDC  Daughter | Forth pregnancy (1 son died), 5 ANC visits  Home birth | From one day after birth  Cold and cough, fever, nose blocked  Difficulty breathing and sucking milk as nose was blocked due to cold. | Newborn illness not yet resolved as cold and cough still giving medicine for but fever and blocked nose gone |
| **NC-5**  Pipariya VDC  Son | Third pregnancy,  1 ANC visit  ultrasound to check the position whether breech or not  Home birth | Baby was not moving after birth  Then baby had difficulty breathing / stuffy nose and shivering | Newborn illness resolved on same day in 6 hours of illness recognition |
| **NC-6**  Babargunj  VDC  Son | Second pregnancy  3 ANC visits  Home birth | After birth**,** Baby was having difficulty breathing made heavy breathing grunting noise and was cold when it was born , baby's limbs went limp, mouth and ears had turned black | Newborn illness resolved within 12 days of illness recognition |
| **NC-7**  Kabilasi VDC  Daughter | Third pregnancy  2 ANC visits  Facility birth – Laxmipur Birthing Center | When child was born had fever  High fever and not breast feeding next day | Newborn illness resolved on 6^th^ day of illness recognition |
| **NC-8**  Babargunj VDC  Daughter | First pregnancy  0 ANC visit  Home birth | The day after the baby was born, the baby had convulsions, difficulty breastfeeding and jaundice | Newborn illness resolved on 3^rd^ day of illness recognition |
| **NC-9**  Barahathawa VDC  Daughter | Third pregnancy (one stillborn)  4 ANC visits  Ultrasound was also done  Home birth | On the 7^th^ day (a week after birth) Baby had cough and cold and due to that had difficulty breathing, looked somewhat green, body was hot | Newborn illness resolved on 6^th^ day of illness recognition |
| **NC-10**  Gadaiya VDC  Daughter | Fifth pregnancy  3 ANC visits  Home birth | Baby was not breathing and body was cold when it was born | Newborn illness was resolved after one month so about day 28 from illness recognition |
